# Evaluating the impact of a tobacco tax increase on smoking cessation outcomes: A seven-year retrospective study from a regional teaching hospital in Taiwan

**DOI:** 10.3934/publichealth.2026001

**Published:** 2026-01-05

**Authors:** Po-Hsun Yang, Yuan-Shan Chien, Dih-Ling Luh

**Affiliations:** 1 The Department of Public Health, Chung Shan Medical University, Taichung, Taiwan; 2 The Department of General Affairs, Taichung Tzu Chi Hospital, Taichung, Taiwan; 3 Disease Control Devision, Changhua County Public Health Bureau, Changhua, Taiwan; 4 The Department of Family and Community Health, Chung Shan Medical University Hospital, Taichung, Taiwan

**Keywords:** tobacco tax, smoking cessation service, smoking cessation treatment, health education consultation, smoking cessation effectiveness, Taiwan

## Abstract

**Objective:**

This study assesses the impact of Taiwan's 2017 tobacco tax increase on smoking cessation outcomes using data from a regional teaching hospital between 2013 and 2019. It focuses on the treatment completion rates for pharmacotherapy and health education interventions.

**Methods:**

This retrospective study involves a secondary data analysis conducted at a regional teaching hospital. The analysis included preventive healthcare and smoking cessation service records from the National Health Insurance Administration database and the hospital's own records. The effectiveness of smoking cessation programs was assessed via follow-up records at three and six months, which were categorized into four groups: failure (smoking-smoking), relapse (not smoking-smoking), delay (smoking-not smoking), and successful cessation (not smoking-not smoking). A multinomial logistic regression model was applied for the multivariate analysis.

**Results:**

No statistically significant difference was observed in the effectiveness of smoking cessation services before and after the tobacco tax increase. However, the participants who failed to complete both the pharmacotherapy and the health education counseling had a markedly higher likelihood of cessation failure compared with those who completed both interventions (*OR* = 4.84, 95% *CI*: 2.69–8.70).

**Conclusion:**

While the 2017 tobacco tax increase did not significantly improve cessation outcomes, service completion was strongly associated with success. Efforts to support full participation in smoking cessation services may enhance the treatment's effectiveness.

## 1. Introduction

Since the release of the first “Smoking and Health” report in 1964 [1] and the World Health Organization's (WHO) 1998 report on tobacco hazards [2], the health risks associated with smoking have become a global public health concern. Smoking not only harms health but also leads to increased smoking-related healthcare expenditures and broader economic burdens [3]. In response, the WHO adopted the Framework Convention on Tobacco Control (WHO FCTC) in 2003, followed by the MPOWER policy package in 2008, which advocates evidence-based strategies such as increased taxation and accessible cessation services [4].

Taiwan launched its “Outpatient Smoking Cessation Treatment Pilot Program” on 2002/09/01. This initiative prioritized pharmacotherapy as the primary intervention and demonstrated its effectiveness in promoting smoking cessation [5]. This pilot program became a regular initiative on 2004/01/01, with expanded subsidies introduced in 2005 and 2006. To further support the participating medical institutions, the National Health Administration (NHA) established the Medical Institution Prevention and Smoking Cessation Service Information System (hereafter, the ‘VPN System’) in 2010, which improved both accessibility and efficiency.

On 2012/03/01, the ‘Second-Generation Smoking Cessation Treatment Pilot Program’ expanded cessation services to outpatient, inpatient, emergency, and community pharmacies. It integrated pharmacotherapy and health education while broadening the participant eligibility criteria.

Past studies have confirmed that increasing tobacco taxes is an effective strategy to reduce tobacco consumption. Raising tobacco taxes not only decreases the likelihood of nonsmokers initiating smoking but also increases the motivation of smokers to quit smoking [6]. In Taiwan, the tobacco pricing strategy was initially introduced through the Tobacco and Alcohol Tax Act [7], which included both tobacco health and welfare surcharges (hereafter referred to as tobacco surcharges) and the tobacco tax. Tobacco surcharges were first implemented in 2002 (set at TWD 5 per pack, later increased to TWD 10 in 2006). In 2007, the principles for setting and allocating tobacco surcharges were formally incorporated into the Tobacco Hazards Prevention Act [8], with the surcharge raised to TWD 20 in the same year. Furthermore, on 2017/06/12, the tobacco tax significantly increased (from TWD 590 per thousand cigarettes to TWD 1590, corresponding to an increase in TWD 20 per pack). During this period, the smoking rates in Taiwan consistently declined, from 27% in 2002 to 22.1% in 2006, 20% in 2009, and 13% in 2018 [5]. There is no long-term observational study in Taiwan regarding the effectiveness of smoking cessation before and after the tobacco tax increases. In 2017, the number of people participating in smoking cessation services at a teaching hospital within central Taiwan increased compared to the previous two years. Therefore, exploring whether participating in smoking cessation services after a tobacco tax increase will lead to successful smoking cessation can help understand the results of policy implementation.

Providing smoking cessation services is another key strategy of the MPOWER framework [4]. In Taiwan, smoking cessation services are generally achieved either without assistance [9] or with interventions and support, including smoking cessation services at medical institutions, cessation hotlines, and health education classes offered by the NHA of the Ministry of Health and Welfare [5].

Despite these initiatives, achieving lasting cessation remains difficult. Tobacco dependence is a chronic condition that often requires repeated treatments [10]. Studies have shown that 61% of individuals who successfully quit smoking relapse within six months [11], and 50%–70% relapse within a year [12],[13]. Smokers may need to attempt to quit at least 30 times before achieving sustained success [14]. Even those diagnosed with severe health issues, such as cancer, chronic obstructive pulmonary disease (COPD), and heart disease, do not experience increased rates of long-term cessation success [15]–[17]. Therefore, both the quit rate and sustained cessation status must be considered when evaluating the effectiveness of smoking cessation programs.

Despite extensive tobacco control efforts, no studies have examined how Taiwan's tobacco tax increases have affected the outcomes of smoking cessation services. Regional hospitals play a pivotal role in providing these services, thereby accounting for nearly 60% of the nation's top-performing cessation programs [5]. In 2019, regional hospitals served over 31,000 individuals across nearly 94,000 visits. Given their central role, understanding the effectiveness of cessation services within regional hospitals is essential.

## 2. Materials and methods

### 2.1. Target population

This study focuses on a regional teaching hospital in central Taiwan and uses two retrospective data sources from the Health Promotion Administration (HPA) medical facility's preventive healthcare and smoking cessation service system and the hospital's records on smoking cessation services from 2010/01/01–2019/12/31. The study was approved by the Research Ethics Committee of Taichung Tzu Chi Hospital, Buddhist Tzu Chi Medical Foundation (approval number REC108–19). Identifiable information was only accessible to authorized researchers, and the statistical analyses were conducted from 2020/08/12–2020/12/31.

A total of 4049 individuals received smoking cessation services. According to the NHA regulations, individuals are eligible for up to two cessation treatments per year (designated as the “first” and “second” treatments). To avoid duplicate entries and repeated measures, this study only included individuals who received their first treatment (*n* = 3551) for the analysis of service volume and annual success rate trends.

**Figure 1.**
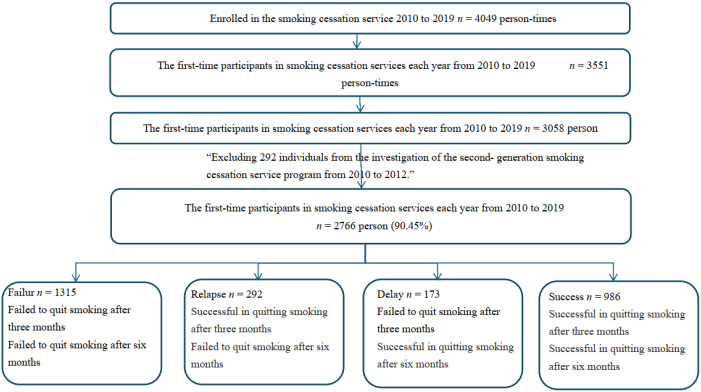
Flowchart of the sample size for data processing and analysis.

To further identify factors associated with smoking cessation success while eliminating the confounding effect of repeated participation, we analyzed data from 3058 individuals who had complete records for their first treatment. To assess post-policy trends following the introduction of the second-generation cessation pilot program (initiated on 2012/03/01, and implemented on 2012/09/01), we excluded 292 cases from 2010 to 2012, which resulted in a final analytic sample of 2766 individuals (90.45%, 2766/3058). Details of the case selection process are shown in Figure 1.

The NHA required participants to be over 18 years of age, insured, and meet one of the following criteria: a Fagerström Nicotine Dependence (FTND) score of 4 or higher or an average of 10 or more cigarettes per day. Additionally, the participants must agree to pay a partial copayment for the cessation pharmacotherapy. Eligible participants could receive up to two 8-week treatment courses per year. Health education consultations, which were delivered face-to-face by contracted educators (group education not allowed), either occurred concurrently with or separately from the pharmacotherapy and needed to be completed within 90 days at the same institution. Follow-up by phone was required at three and six months to track the cessation rates, with results recorded in the VPN system.

Starting in 2018, the NHA implemented telephone checks and defined “unable to contact” as a failure to quit smoking. The results of these checks were recorded in the VPN system as the final smoking cessation outcomes. “Unable to contact” refers to cases where there is no phone number or the number provided is not in use, which led to some successful cessation records being marked as failures.

Therefore, this study excluded the sample size from 2018 to avoid false errors affecting the results, and the results of the two tests are presented in Table 1.

### 2.2. Variables definition

#### 2.2.1. Dependent variables

Cessation status was recorded by cessation coordinators through phone interviews at three- and six-month post treatment, where the participants were asked ‘Have you smoked in the past 7 days?’ Responses of ‘smoked’ are classified as cessation failures, whereas ‘not smoked’ indicates successful cessation.

For the first research objective, the success rate was calculated by dividing the number of registered participants (numerators) by the number of successfully contacted individuals (denominators), with unsuccessful contacts counted as failures.

For the second and third research objectives, ‘cessation continuity’ was classified into four categories based on the follow-up assessments at three and 6 months: (1) failure: failure at both 3 and 6 months; (2) relapse: success at 3 months but failure at 6 months; (3) delay: failure at 3 months but success at 6 months; and (4) success: success at both 3 and 6 months.

In this study, the dependent variable—smoking cessation outcome—was modeled with successful cessation as the reference category. Emphasizing failure as the outcome enables a more precise evaluation of intervention strategies. By identifying the determinants of unsuccessful cessation participants, tailored programs can be developed to address specific barriers and ultimately improve the overall effectiveness.

#### 2.2.2. Independent variables

Tobacco Tax Increase: The participants were categorized into two groups based on the year of the tobacco tax increase (2017/06/12): pre-tax and post-tax increase.

Completion of Cessation Treatment: The second-generation cessation program included both pharmacotherapy and health education consultations, with no requirement for full course completion. Completion status was categorized as follows: first, both pharmacotherapy and education completed; second, only pharmacotherapy completed; third, only education completed; and fourth, neither completed.

Other factors: Additional independent variables included demographic characteristics, smoking history, and cessation service records. The demographic variables included gender (male, female), age at participation (categorized as under 40, 40–64, and 65 years or older), education level (below junior high school, high school, or above college), and marital status (married, single, other and no response). The medical history included the presence of any of the following conditions: brain diseases, cardiovascular diseases, diabetes, kidney diseases, liver diseases, cancer, or mental disorders. Health behaviors included alcohol consumption and betel nut chewing, categorized as ‘yes’ or ‘no/no response’. The smoking history included smoking duration (under 20 years, over 20 years), smoking quantity (0–10, 11–20, 21–30, >30 cigarettes per day), and nicotine dependence (FTND scores of 0–7, 8–10). The cessation process factors included referral source (outpatient, inpatient) and cessation-related side effects (present/absent).

### 2.3. Data processing and statistical analysis

**Figure 2.**
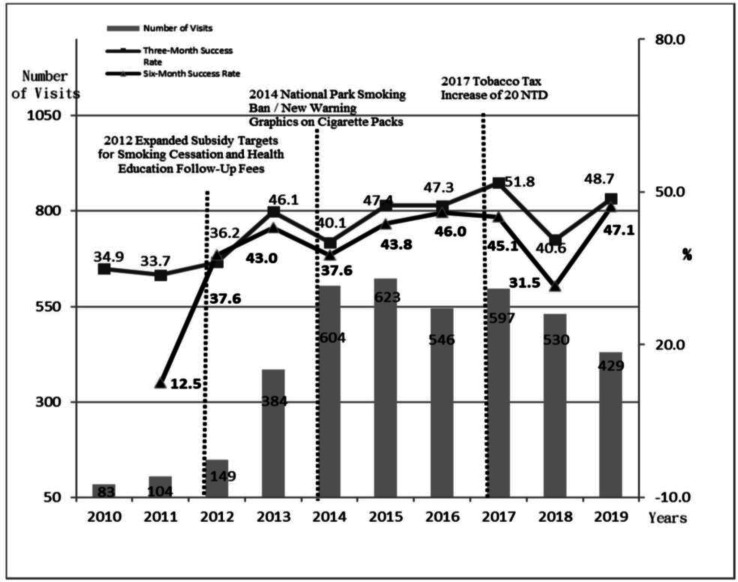
Annual distribution of service visits and smoking cessation success rates at a smoking cessation clinic in a regional hospital in central Taiwan.

**Figure 3.**
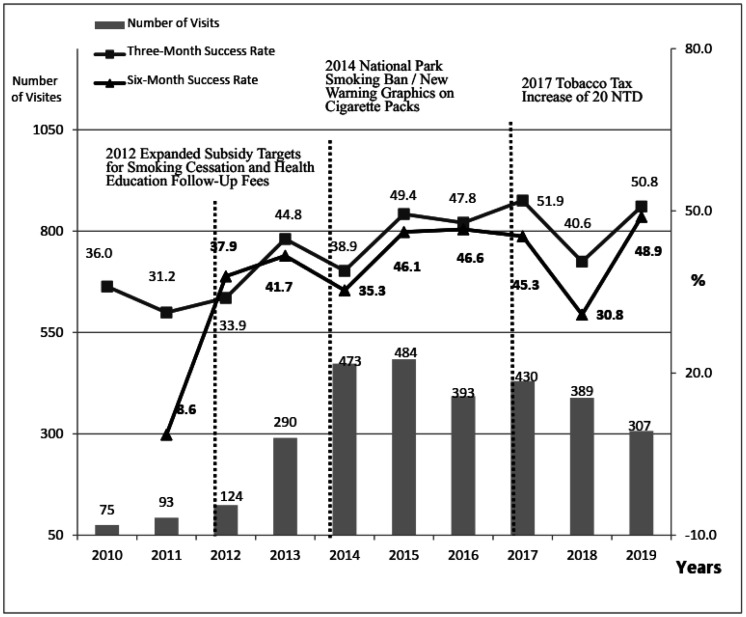
Annual distribution of first-time service users and smoking cessation success rates at a smoking cessation clinic in a regional hospital in central Taiwan.

To analyze the impact of the 2017/06/12 tobacco tax increase on smoking cessation effectiveness, the data was processed in two ways. First, the annual trends in participation numbers and success rates were described, thereby presenting participation as total visits (allowing multiple participation per individual) (Figure 2) and first-time participants (one record per individual) (Figure 3). This study used chi-square tests to examine the correlations between pre- and post-tax increases and the independent variables affecting the effectiveness of smoking cessation services.

Second, individual-level analyses were conducted to examine the distribution and relationships of personal variables (demographics, smoking status, service usage, and completion status) in relation to cessation continuity. Cessation continuity, the key effectiveness indicator (failure, relapse, delay, success), was analyzed via a binary logistic regression model to assess the impact of a tobacco tax increase and cessation services on the cessation outcomes. Finally, a multiple logistic regression model was used to analyze the association between categorical variables and successful quitting.

All statistical analyses were performed using SAS 9.4 FOUNDATION for Windows V6.2, with a significance level (*α*) set at 0.05.

## 3. Results

Figures 2 and 3 illustrate trends in the participation rates and success rates for smoking cessation services from 2010–2019. Figure 2 presents the raw participation data, whereas Figure 3 shows the annual trend for first-time participants.

The quantity of cessation services (gray bars) significantly increased starting in 2013, following the expansion of subsidies for the second-generation cessation program, with increases of 157.7% and 133.9% compared with those in 2012 (data not shown). The upward trend continued in 2014, after smoking bans in national parks and new warning graphics on cigarette packages, which resulted in increases of 57.3% and 63.1%, respectively, compared with 2013 (not shown in the table). Afterward, service usage slightly declined, with a small increase in 2017, and the year of the tobacco tax increased, followed by a gradual decrease in subsequent years.

In terms of success rates, the highest three-month cessation success rate (square lines) was recorded in 2017 (51.8% for all participants, 51.9% for first-time participants). The highest six-month success rate (triangular lines) was recorded in 2019 (47.1% for all participants, 48.9% for first-time participants).

In 2018, the cessation success rate was notably lower, which was attributed to the NHA's phone follow-up policy, where the participants who could not be contacted were classified as cessation failures. This led to some successful cessations being misclassified as failures. Consequently, hospitals strictly enforced the requirement for participants to provide contact numbers where they could be directly reached. The data show that the cessation success rate in 2019 returned to a level comparable to that in 2017.

To assess the cessation outcomes after the introduction of the second-generation cessation policy (effective 2012/01/09), we analyzed data for 2766 first-time participants between 2013 and 2019, excluding data from 2010 to 2012 (Table 1).

Following the NT$20 tobacco tax increase on 12/06/2017, the participation in cessation services was slightly greater in 2017 than in 2016 but lower than in previous years, thus showing a gradual decline. The cessation effectiveness in 2018 deviated from trends observed in other years. Excluding the 2018 data, the cessation failure rates in 2017 and 2019 were slightly lower than those in the years prior to the tax increase, whereas the continued cessation rates were slightly higher.

A chi-square test that examined the relationship between the tax increase and cessation effectiveness (the results are shown in table 1) revealed that when the 2018 data were included, the post-tax failure rate was slightly higher than the pretax rate. However, after excluding the 2018 data, the post-tax failure rate was lower, with a significantly higher rate of continued cessation.

To avoid confounding the results and to simplify interpretation, the 2018 data were excluded from subsequent analyses.

**Table 1.** Smoking cessation status of the research subjects by policy year (*n* = 2766).

Variables	Overall		Effectiveness of smoking cessation services								*χ^2^*	*p*-value
			Failure		Relapse		Delay		Success			
	*n*	%	*n*	%	*n*	%	*n*	%	*n*	%		

Years\N	2766	100	1315	47.5	292	10.6	173	6.3	986	35.7		
2013	290	10.5	119	41.0	50	17.2	41	14.1	80	27.6	117.5	<0.0001***
2014	473	17.1	251	53.1	55	11.6	38	8.0	129	27.3		
2015	484	17.5	219	45.3	42	8.7	26	5.4	197	40.7		
2016	393	14.2	181	46.1	29	7.4	24	6.1	159	40.5		
2017	430	15.6	189	44.0	46	10.7	18	4.2	177	41.2		
2018	389	14.1	221	56.8	48	12.3	10	2.6	110	28.3		
2019	307	11.1	135	44.0	22	7.2	16	5.2	134	43.7		

**Tobacco tax increase (with 2017/06/ 12, as the statistical date before and after the increase, *n* = 2766)**												
Variables	Overall		Effectiveness of smoking cessation services								*χ^2^*	*p*-value
			Failure		Relapse		Delay		Success			
	*n*	%	*n*	%	*n*	%	*n*	%	*n*	%		

Before	1839	66.5	852	46.3	200	10.9	139	7.6	648	35.2	17.4	0.0006**
After	927	33.5	463	50.0	92	9.9	34	3.7	338	36.5		

**Tobacco tax increase (with 2017/06/ 12, as the statistical date before and after the increase, excluding 2018, *n* = 2377)**												
Variables	Overall		Effectiveness of smoking cessation services								*χ^2^*	*p*-value
			Failure		Relapse		Delay		Success			
	*n*	%	*n*	%	*n*	%	*n*	%	*n*	%		

Before	1839	77.4	852	46.3	200	10.9	139	7.6	648	35.2	14.7	0.0021**
After	538	22.6	242	45.0	44	8.2	24	4.5	228	42.4		

Note: Statistical significance: * *p < 0.05*, ** *p < 0.01*, *** *p < 0.001*.

Table 2 presents the cessation service completion status and its distribution on the basis of effectiveness. Only 3.62% of the participants fully completed both the eight-week medication treatment and the health education consultations. A chi-square test revealed a significant association between completion status and success rates: the participants who completed both pharmacotherapy and education had the highest continued cessation success rate (54.65%), whereas those who did not complete either had the lowest (35.22%).

Additionally, other independent variables related to cessation effectiveness are presented in Table 2. Most participants were male (89.19%) and married (48.04%). The majority had an education level up to junior high school (38.49%) and over 20 years of smoking experience (80.82%). Regarding medical history and health behaviors, 68.36% reported having at least one medical condition, 77.11% experienced no cessation-related side effects, and 73.92% did not consume alcohol. In the multinomial logistic regression, several demographic variables showed significant correlations. Participants aged ≤40 years accounted for 22.47%, those aged 40–64 years accounted for 63.19%, and those aged ≥65 years accounted for 14.35%. Betel nut chewing was reported by 9.55% of the participants, while 90.45% did not chew or did not respond. The number of cigarettes smoked per day was distributed as follows: 0–10 (24.91%), 10–20 (42.24%), 20–30 (14.35%), and >30 (18.51%). Based on the FTND scores, 80.69% scored 0–7 points and 19.31% scored 8–10 points. The referral sources included inpatient transfers (61.17%) and outpatient clinics (38.83%). Among the participants, 48.42% received both pharmacotherapy and health education counseling, 48.04% only received counseling, and 3.53% only received pharmacotherapy.

**Table 2.** Relationships between smoking cessation service effectiveness and participation, excluding 2018 (*n* = 2377).

Variables	Overall		Effectiveness of smoking cessation services								*χ^2^*	*p*-value
			Failure		Relapse		Delay		Success			
	*N*	%	*N*	%	*N*	%	*N*	%	*N*	%		
	2377	100	1094	46.0	244	10.3	163	6.9	876	36.9		
Tobacco tax increase												
Before the increase	1839	77.37	852	46.3	200	10.9	139	7.6	648	35.2	14.7	0.0021**
After the increase	538	22.63	242	45.0	44	8.2	24	4.5	228	42.4		
Duration of treatment category*												
P+, C+	86	3.62	18	20.9	14	16.3	7	8.1	47	54.7	39.1	<0.0001***
P+, C-	193	8.12	80	41.5	19	9.8	11	5.7	83	43.0		
P-, C+	119	5.01	41	34.5	20	16.8	9	7.6	49	41.2		
P-, C-	1979	83.26	955	48.3	191	9.7	136	6.9	697	35.2		
Gender												
Male	2120	89.19	965	45.5	200	10.4	146	6.9	789	37.2	2.1	0.5603
Female	257	10.81	129	50.2	24	9.3	17	6.6	87	33.9		
Age												
40 or below	534	22.47	299	56.0	43	8.1	41	7.7	151	28.3	78.0	<0.0001***
40–64	1502	63.19	696	46.3	159	10.6	106	7.1	541	36.0		
65 or above	341	14.35	99	29.0	42	12.3	16	4.7	184	54.0		
Education												
Junior high school or below	616	25.92	275	44.6	72	11.7	42	6.8	227	36.9	17.8	0.038*
Senior high school	581	24.44	292	50.3	69	11.9	36	6.2	184	31.7		
College or above	334	14.05	157	47.0	32	9.6	25	7.5	120	35.9		
No response	846	35.59	370	43.7	71	8.4	60	7.1	345	40.5		
Marital status												
Single	357	15.02	188	52.1	45	12.6	23	6.4	103	28.9	17.1	0.009**
Married	1142	48.04	508	44.5	126	11.0	79	6.9	429	37.6		
Other/no response	878	36.94	400	45.6	73	8.3	61	7.0	344	39.2		
Disease												
No/no response	747	31.43	362	48.5	59	7.9	63	8.4	263	35.2	12.1	0.0071**
Yes	1630	68.57	732	44.9	185	11.4	100	6.1	613	34.6		
Alcohol consumption												
No/no response	1757	73.92	779	44.3	180	10.2	115	6.6	683	38.9	12.6	0.0056**
Yes	620	26.08	315	50.8	64	10.3	48	7.7	193	31.1		
Betel nut chewing												
No/no response	2150	90.45	960	44.7	220	10.2	147	6.8	823	38.3	21.7	<0.0001***
Yes	227	9.55	134	59.0	24	10.6	16	7.1	53	23.4		
Daily cigarette consumption												
0–10	592	24.91	184	31.1	56	9.5	31	5.2	321	54.2	151.2	<0.0001***
10–20	1004	42.24	461	45.9	96	9.6	77	7.7	370	36.9		
20–30	341	14.35	188	55.1	52	15.3	20	5.9	81	23.8		
>30	440	18.51	261	59.3	40	9.1	35	8.0	104	23.6		
Smoking years												
<20 years	456	19.18	231	50.7	33	7.2	40	8.8	152	33.3	12.6	0.0056**
>20 years	1921	80.82	863	44.9	211	11.0	123	6.4	724	37.7		
FTND score												
0–7points	1918	80.69	820	42.8	198	10.3	121	6.3	779	40.6	65.6	<0.0001***
8–10points	459	19.31	274	59.7	46	10.0	42	9.2	79	21.1		
Referral source												
inpatient	1454	61.17	601	41.3	168	11.6	99	6.8	586	40.3	36.1	<0.0001***
outpatient	923	38.83	493	53.4	76	8.2	64	6.9	290	31.4		
Smoking cessation service category												
Pharmacotherapy	84	3.53	44	52.4	7	8.3	12	14.3	21	25.0	53.6	<0.0001***
Health education counseling	1142	48.04	461	40.4	124	10.9	62	5.4	495	43.4		
Both	1151	48.42	589	51.2	113	9.8	89	7.7	360	31.3		
Smoking cessation side effects												
No/no response	1833	77.11	869	47.4	183	10.0	124	6.8	657	3.5.84	6.3	0.0992
Yes	544	22.89	225	41.4	61	11.2	39	7.2	219	40.3		
Completion of pharmacotherapy												
Completed	279	11.74	98	35.1	33	11.8	18	6.5	130	45.7	95.5	<0.0001***
Uncompleted	956	40.22	535	56.0	87	9.1	83	8.7	251	26.3		
Health education counseling	1142	48.04	461	40.4	124	10.9	62	5.4	495	43.4		
Duration of health education counseling												
Finished	205	8.62	59	28.8	34	16.6	16	7.8	96	46.8	40.2	<0.0001***
Unfinished	2088	87.84	991	47.5	203	9.7	135	6.5	759	36.4		
Pharmacotherapy	84	3.53	44	52.4	7	8.3	12	14.3	21	25.0		

Note: * P = pharmacotherapy; C = health education counseling; + finished; - unfinished. Statistical significance: * *p* < 0.05, ** *p* < 0.01, *** *p* < 0.001.

This study revealed no statistically significant relationship between the tobacco tax increase and the cessation service effectiveness after adjustments were made for other variables (Table 3).

The completion status was significantly associated with cessation success. The participants who completed both pharmacotherapy and education had the lowest odds of failure, whereas those who completed only pharmacotherapy or education had 2.29 (*OR* = 2.29, 95% *CI*: 1.19, 4.42) and 2.79 (*OR* = 2.79, 95% *CI*: 1.35, 5.77) times greater odds of cessation failure, respectively. The participants who completed neither had 4.84 times (*OR* = 4.84, 95% *CI*: 2.69, 8.70) higher odds of failure.

Other key findings include the following: (1) younger participants were more likely to fail or experience delayed cessation than were those aged 65 and older; (2) betel nut chewers had higher failure rates; (3) a higher daily cigarette consumption was associated with a greater likelihood of cessation failure than was lower consumption (0–10 cigarettes); (4) the participants with higher nicotine dependence (FTND scores of 8–10) were more likely to fail; and (5) inpatient referrals were associated with high failure rates than were outpatient referrals.

**Table 3.** Factors associated with continued smoking cessation status: A multinomial logistic regression analysis (*n* = 2377).

Variables	**Smoking Cessation Status**					
	Failure/Success		Relapse/Success		Delay/Success	
	*OR*	95% *CI*	*OR*	95% *CI*	*OR*	95% *CI*
Tobacco tax increase						
Before the increase	1		1		1	
After the increase	1.08	0.85–1.39	0.75	0.50–1.13	0.62	0.38–1.03
Gender						
Female	1					
Male	0.77	0.56–1.05	0.9	0.55–1.47	0.99	0.56–1.74
Age						
65 or above	1		1		1	
40–64	2.06	1.54–2.75	1.09	0.73–1.64	1.94	1.09–3.44
40 or below	3.73	2.42–5.76	1.27	0.67–2.45	2.13	0.96–4.74
Education						
Junior high school or below	1		1		1	
Senior high school	0.96	0.73–1.27	1.13	0.75–1.70	0.78	0.47–1.30
College or above	0.75	0.53–1.04	0.82	0.49–1.37	0.76	0.42–1.37
No response	0.72	0.51–1.02	0.85	0.51–1.41	0.82	0.44–1.49
Marital status						
Married	1		1		1	
Single	1.21	0.88–1.64	1.47	0.95–2.29	0.9	0.52–1.57
Other/no response	1.18	0.87–1.60	0.94	0.60–1.50	1.02	0.60–1.75
Disease						
No	1		1		1	
Yes	1.01	0.78–1.31	1.27	0.84–1.91	0.8	0.51–1.26
Alcohol consumption						
No/no response	1		1		1	
Yes	1.01	0.83–1.34	0.95	0.66–1.36	1.2	0.80–1.82
Betel nut chewing						
No/no response	1		1		1	
Yes	1.55	1.08–2.22	1.33	0.78–2.27	1.21	0.65–2.26
Daily cigarette consumption						
0–10	1		1		1	
10–20	1.9	1.50–2.40	1.45	1.00–2.09	1.88	1.19–2.95
20–30	3.25	2.29–4.59	3.52	2.16–5.73	1.6	0.82–3.14
>30	2.88	1.91–4.34	1.7	0.90–3.18	1.44	0.68–3.05
Smoking years						
<20 years	1		1		1	
>20 years	1.39	0.98–1.98	1.6	0.90–2.84	0.75	0.41–1.36
FTND score						
0–7 points	1					
8–10 points	1.49	1.02–2.15	1.26	0.73–2.18	2.62	1.40–4.93
Referral source						
outpatient	1		1		1	
inpatient	1.66	1.35–2.05	0.92	0.66–1.29	1.29	0.89–1.88
Smoking cessation side effects						
No/no response	1					
Yes	0.96	0.76–1.22	1.09	0.77–1.55	1.1	0.73–1.66
Duration of treatment category*						
P+, C+	1		1		1	
P+, C-	2.29	1.19–4.42	0.88	0.39–1.97	0.73	0.26–2.07
P-, C+	2.79	1.35–5.77	1.43	0.63–3.29	1.44	0.48–4.34
P-, C-	4.84	2.69–8.70	1.15	0.60–2.22	1.49	0.64–3.47

Note: * P = pharmacotherapy; C = health education counseling; + finished; - unfinished.

## 4. Discussion

This study analyzed seven years of smoking cessation data from a regional teaching hospital in Taiwan, and yielded three main findings:

- first, after we adjusted for key variables, the 2017 tobacco tax increase had no significant effect on smoking cessation outcomes;
- second, the participants who did not complete the full cessation program, which included eight weeks of pharmacotherapy and health education, were significantly more likely to relapse at both three and six months; and
- third, inpatient referrals, older participants, those with a betel nut chewing habit, those with higher smoking volumes, and those with greater nicotine dependence had a greater likelihood of failure.

### 4.1. Tax rise, no quit link

The 2013 tobacco tax increase had no significant effect on the smoking cessation outcomes at this regional teaching hospital, which contrasts with findings from studies conducted abroad. For example, research in the United States revealed that a tobacco tax increase significantly increased a smoker's willingness to quit [18],[19] and studies in Japan reported a marked increase in the smoking cessation rate following a tax hike [20]. Possible reasons for the lack of impact are described below.

The tax increase may have been insufficient to effectively motivate smokers to quit [21], potentially leading to relapse among those who initially quit [20]. For example, a 2002 study on the first tobacco tax levy in Taiwan reported that a price increase of TWD 5 per pack had only a limited effect on reducing tobacco use [22]. In his study, Richard Felsinger encouraged countries world-wide to use price policies and taxation more intensively in order to effectively reduce the smoking rates [23]. Following the 2017 tax adjustment, the price for the cheapest cigarette brand, Chang Shou, was TWD 90 (approximately USD 2.73), with TWD 51.8 (USD 1.57) attributed to taxes, thus accounting for 57.56% of the total price. This relatively low increase may explain why the tax adjustment did not have a noticeable effect on smoking cessation.

Smokers may have switched to cheaper brands or sought tobacco from alternative lower-cost sources, thus mitigating the impact of the tax increase. While tobacco companies offer a range of cigarette prices [24] and smokers may access cheaper alternatives through informal markets [25], this study could not confirm this effect on the basis of secondary data analysis.

The impact of price increases on smoking cessation may vary across demographic groups. Research suggests that higher tobacco prices are more effective in encouraging cessation among older adults, particularly those aged 65 and older [26],[27]. In this study, older smokers were more successful in quitting than their younger counterparts, which may reflect some influence of the tax increase.

The effects of price hikes on smoking may be short-lived. A study in California reported that the significant increase in cessation rates following a 95-cent price hike in 1998 only lasted four months [28].

The impact of price increases on smoking behaviors may not be solely captured through hospital-based cessation services. Smoking cessation services provided by hospitals represent just one method of quitting, and smokers may choose alternative approaches, such as quitting independently, attending community-based cessation programs, or consulting pharmacies for cessation medications. Consequently, the findings of this study reflect the lack of impact of the tax increase on the smoking cessation services offered at a specific regional teaching hospital but do not necessarily represent the broader effects of the tobacco tax increase on overall smoking cessation behaviors.

### 4.2. Smoking cessation service completion impact

This study revealed that those who did not complete the recommended number of weeks of cessation services had a greater likelihood of relapses, which is consistent with both domestic and international research. In Taiwan, studies have shown that participants who attend more sessions and who adhere to longer pharmacotherapy treatments have a lower risk of ‘failure to quit or relapse’ [29]. Additionally, those who continued treatment with medications such as varenicline had a significantly higher success rate than for those who attend fewer than three times [30]–[32].

Similar findings have been reported abroad, where using pharmacotherapy for at least five weeks [33],[34] and receiving more than eight health education consultations [35] were associated with a greater likelihood of successful cessation. These findings underscore the importance of completing cessation services to achieve positive outcomes.

The mechanisms behind successful health education interventions include enhancing the motivation to quit, increasing self-efficacy, and providing social support during the cessation process [36]. Medication-assisted cessation works by substituting nicotine and blocking its binding to receptors (α4 and β2), thus suppressing nicotine dependence and aiding cessation [37]. Factors influencing the success of cessation interventions include the following: (1) quitting due to withdrawal symptoms [38],[39]; (2) the inability to complete the eight-week regimen due to insufficient personal determination or willpower [40]; and (3) social and environmental barriers, such as peer smoking temptation [41],[42].

### 4.3. Other related factors to the smoking cessation services

This study identified several factors affecting the effectiveness of smoking cessation services at regional teaching hospitals, including age, betel nut chewing, smoking volume, nicotine dependence, and the referral source.

No significant relationship was found between sex and cessation success, which is consistent with mixed findings in previous studies. Some studies reported that men had significantly higher quit rates than women [43]–[45], whereas others reported no significant relationship between gender and cessation success [46] or that women had significantly higher quit rates than men [47],[48]. However, age was positively correlated with cessation success, with older individuals having higher success rates. Previous studies have produced conflicting results on age, with some suggesting that older individuals are less likely to ‘fail to quit or relapse’ [29],[49]. Other studies have shown that both younger and older individuals may be more likely to quit than middle-aged individuals [48],[50], whereas some studies have indicated that younger individuals might be more likely to quit than older individuals [51].

This study revealed that betel nut chewers were more likely to relapse, which is consistent with prior findings [52]. While alcohol consumption did not affect the cessation outcomes in this study, which is consistent with prior findings [53], some studies abroad linked daily alcohol consumption to lower cessation success [54].

Both daily smoking volume and nicotine dependence were associated with a lower cessation success. Similar findings have been reported in both domestic and international studies, which suggest that a longer smoking history, higher daily consumption, and greater nicotine dependence are associated with lower cessation success rates [49],[54]–[57]. Additionally, inpatient referrals had lower success rates, which is consistent with findings from domestic [58] and international studies [59],[60]. Continuous cessation support following hospital discharge could improve the success rates by providing sustained intervention and guidance.

## 5. Study limitations and future research directions

This study has several limitations. First, the analysis was based on data from a single regional teaching hospital in central Taiwan, and the results may not be generalizable to other hospitals or nationwide trends. Second, the smoking cessation outcomes were only assessed at 3 and 6 months, thus limiting the ability to evaluate long-term abstinence and relapse patterns. Third, the lack of information on individuals lost to follow-up and the reasons for discontinuing outpatient cessation services restricted further analyses. Fourth, the study solely focused on smokers who sought outpatient cessation services, which may represent only one subset of the broader population affected by the tobacco tax increase; individuals who quit independently or through alternative methods were not captured. Finally, during the study period (2002–2019), Taiwan's tobacco control policies underwent multiple revisions, including expanded smoking restrictions and updated warning labels on cigarette packages, which may have influenced a smoker's motivation to quit and contributed to the observed outcomes.

Future studies should utilize nationwide data sets to evaluate the population-wide impact of tobacco tax policies and extend the follow-up periods to 12 months or longer to better capture long-term cessation success and relapse patterns. Moreover, future research is needed to examine the effects of specific components of cessation programs, such as medication types and the frequency of health education sessions. In addition, exploring alternative or informal cessation methods used outside of medical institutions could provide a more comprehensive understanding of quitting behaviors. Finally, subgroup analyses by age, income, education level, and urban–rural residence are recommended to identify the differential impacts of tobacco taxes across diverse populations.

## 6. Conclusion

This seven-year analysis of smoking cessation services at a regional teaching hospital in Taiwan found no statistically significant change in the cessation success rates after the 2017 tobacco tax increase. These results suggest that when governments cannot substantially raise tobacco prices in one step, taxation alone may have a limited impact on achieving a smoke-free society. The completion of cessation services—especially adherence to both pharmacotherapy and counseling—was strongly associated with better outcomes. To improve the effectiveness, healthcare providers should encourage full-course participation and adopt flexible, patient-centered follow-up strategies. Although no further tax increases have been proposed since this study period, the NHA has emphasized treatment continuity. Since January 2023, eligible individuals may receive a third course of cessation treatment within 90 days of the previous one if they show meaningful smoking reduction (e.g., from 30 to 10 cigarettes per day). Eligibility is no longer limited by daily cigarette use or dependence score, thus reflecting a policy shift toward continuous, individualized cessation support.

## Use of AI tools declaration

The authors declare they have not used Artificial Intelligence (AI) tools in the creation of this article.
